# Effectiveness of a psycho-educational group program for major depression in primary care: a randomized controlled trial

**DOI:** 10.1186/1471-244X-12-230

**Published:** 2012-12-18

**Authors:** Rocío Casañas, Rosa Catalán, Jose Luis del Val, Jordi Real, Sergi Valero, Miquel Casas

**Affiliations:** 1 Research Department. Centre Higiene Mental (CHM) Les Corts, c/ Numancia, 103-105, Bajos, 08029, Barcelona, Spain; 2 Psychiatry and Legal Medicine Department, Universidad Autónoma de Barcelona, Barcelona, Spain; 3 Barcelona Research Support Unit in Primary Care. IDIAP Jordi Gol, Catalan Institute of Health, Barcelona, Spain; 4 Clinical Institute of Neurosciences (ICN), Hospital Clinic, C/ Villarroel 170, 08036, Barcelona, Spain; 5 Department of Psychiatry and Clinical Psychobiology, University of Barcelona, Barcelona, Spain; 6 Institut d'Investigacions Biomèdiques August Pi i Sunyer (IDIBAPS), Barcelona, Spain; 7 Centro de Investigación Biomédica en Red de Salud Mental (CIBERSAM), Madrid, Spain; 8 Department of Psychiatry, Hospital Universitari Vall d’Hebron, C/Passeig de la Vall d’Hebron, 119-129, 08035, Barcelona, Spain

**Keywords:** Depression, Primary Care, Psycho-educational, Clinical trials, Nurse

## Abstract

**Background:**

Studies show the effectiveness of group psychoeducation in reducing symptoms in people with depression. However, few controlled studies that have included aspects of personal care and healthy lifestyle (diet, physical exercise, sleep) together with cognitive-behavioral techniques in psychoeducation are proven to be effective.

The objective of this study is to assess the effectiveness of a psychoeducational program, which includes aspects of personal care and healthy lifestyle, in patients with mild/moderate depression symptoms in Primary Care (PC).

**Methods:**

In a randomized, controlled trial, 246 participants over 20 years old with ICD-10 major depression were recruited through nurses/general practitioners at 12 urban Primary Care Centers (PCCs) in Barcelona. The intervention group (IG) (n=119) received a group psychoeducational program (12 weekly, 1.5 h sessions led by two nurses) and the control group (CG) (n=112) received usual care. Patients were assessed at baseline and at, 3, 6 and 9 months. The main outcome measures were the BDI, EQ-5D and remission based upon the BDI.

**Results:**

231 randomized patients were included, of whom 85 had mild depression and 146 moderate depression. The analyses showed significant differences between groups in relation to remission of symptoms, especially in the mild depression group with a high rate of 57% (p=0.009) at post-treatment and 65% (p=0.006) at 9 month follow up, and only showed significant differences on the BDI at post-treatment (p=0.016; effect size Cohen’s d’=.51) and at 6 and 9 month follow-up (p= 0.048; d’=.44).

In the overall and moderate sample, the analyses only showed significant differences between groups on the BDI at post-treatment, p=0.02 (d’=.29) and p=0.010 (d’=.47), respectively.

The psychoeducation group improved significantly on the EQ-5D at short and long-term.

**Conclusions:**

This psychoeducational intervention is a short and long-term effective treatment for patients with mild depression symptoms. It results in a high remission rate, is recommended in PC and can be carried out by nurses with previous training. In moderate patients, group psychoeducation is effective in the short-term.

**Trial registration:**

Clinical Trials.gov identifier NCT00841737

## Background

As depressive disorders are major public health problems with a prevalence of major depression in Europe of 8.56% [1] and, strikingly, 5-16% in primary care patients, they are the third leading cause of consultation in primary health care [2-4].

The impact of depressive symptoms on quality of life (QoL) has already been shown in a community population [5]. The impact of depression on patients’ well-being and QoL has been shown to be equal to or greater than several other major chronic medical conditions such as diabetes mellitus, heart disease and arthritis [6-8].

Depression has been associated with greater morbidity, mortality, health care utilization and health care costs [9,10]. The economic costs of depression have doubled in the last ten years, mainly due to an increase in indirect costs from loss of productivity [11].

There are a wide variety of psychological interventions for treating depression [12] and one which has been shown to be effective in treating depression is psychoeducation [12,13]. A meta-analysis indicates that psychoeducation has an effect size that is comparable with other modalities of treatment for depression [14]. With regard to the management of mild to moderate depression symptoms, psychoeducation is an effective therapy in the treatment of depression in adults [13,15] as it reduces the depression symptoms and can prevent depression in primary care patients [16-18].

Most international clinical practice guidelines (CPG) for the management of depression recommend psychoeducational interventions and brief psychotherapies as a first step in the treatment protocol [15,19,20].

In this case, when we talk about psychoeducation, we refer to group psychoeducation which is offered to people with a mental disorder to help them understand and manage their disease. This is done by reinforcing patients’ resources and skills so that they are better able to cope with their situation, prevent relapse and contribute to their own health and welfare. The educational intervention was based on the patients’ active involvement in the analysis of their situation and its solution. As such, it includes psychotherapeutic techniques (behavioral activation, cognitive behavioral therapy and problem solving) and homework tasks. At the group level, information is provided to several patients about their condition and they have the chance to exchange ideas about their experiences; creating a space for mutual help group [21-23].

Psychoeducation has been used in health-care and community settings and seems effective in prevention and quality improvement programs in US primary care [18,24,25]. In Europe, this psychoeducational intervention has proved to be effective in reducing depression symptoms in mild and moderate depression in the short term [26,27] and long-term [28], and has shown it can be carried out by community nurses with previous training [24,27,29,30].

The majority of studies which have assessed the effectiveness of psychoeducation have used “Coping with depression (CWD)” or a version of it. The CWD is a cognitive, psychoeducative, behavioral intervention based on the theory of depression and social learning (Teri & Lewinsohn, 1986) [31] which aims to improve self-esteem levels and social support, and develop/improve those skills which have been found to help in the prevention of depression from its onset: social skills, activity level management (pleasurable activities) and management of depressive thought.

There are other psychoeducational formats such as a psyco-educational cognitive workshops [32], cognitive bibliotherapy [33], education and group counselling [34], and some psychoeducational interventions as well as computerized cognitive behavioral therapy [35-37] and psychoeducation through a website [38] which included aspects of the healthy lifestyle recommended in their program.

To date, there is evidence of the effectiveness of psychoeducational intervention in the Primary Care depressive population [26,27], although there are no studies that have recorded important aspects of patients’ personal care (diet, physical activity, sleep hygiene, information on the importance of therapeutic adherence and the side-effects of pharmacological treatment) and the identification and management of depression symptoms within the psychoeducational group intervention; aspects which have already been shown to aid recovery in these patients [15,39]. This new psychoeducational group intervention format is based on three fundamental care aspects for the patient with depression: improving knowledge of the disorder, promoting a healthy life-style and acquisition of habits which are beneficial to health, and the development of resources to cope with critical situations (behavioral activation techniques, cognitive behavioral therapy, problem-solving and breathing-relaxation).

The main objective of the study is to evaluate the effectiveness of this intervention through the rate of remission in the study sample post-intervention and at 6 and 9-month follow-up and, to determine whether the improvement in depression symptoms is associated with an improvement in quality of life at post-treatment and at follow-up.

As secondary objectives, we were interested in analyzing which population most benefits from this intervention; the population with “mild” or “moderate” symptoms.

## Methods

Randomized controlled trial, open parallel-group was conducted between December, 2008, and April, 2010, in primary care centers in Barcelona, Spain.

### Participants

A total of 246 participants were recruited by general practitioners (GPs) and nurses between December, 2008, and March, 2009, in 12 primary care centers (PCC) in the Barcelona urban area. These PCC participants had varying socio-demographic and economic resources.

Inclusion criteria were: a) patients older than 20 years of both sexes; b) diagnosed with a major depressive disorder according to the International Classification of Disease 10^th^ revision (ICD-10) [40]; c) having mild to moderate symptoms according to the Beck Depression Inventory (BDI ≥10 and <30) and d) provision of signed informed consent.

Exclusion criteria were: a) patients with other diagnosed associated psychiatric disorders (including personality disorders and drug and alcohol abuse); b) current presence of suicidal ideation or suicide attempts; c) using secondary mental health services; d) patients with acute or terminal medical illness; e) inability to speak and understand Spanish and/or Catalan language; f) sensory or cognitive disabilities; g) illiteracy; h) temporary residents or i) non-provision of consent. Concurrent treatment with antidepressants was not an exclusion criterion but patients taking antidepressant medication could not have changed their treatment during the previous month.

Ethical approval was granted by the Jordi Gol i Gurina Foundation. Informed consent was obtained from all participants prior to their involvement in the study.

### Procedure

#### Recruitment of participants

Nurses and GPs at each PCC were responsible for identifying patients with a possible diagnosis of depression. Once detected, the GPs were responsible for diagnosing depression disorder according to the ICD-10. Participants who met BDI criteria for mild or moderate depression (a score of BDI ≥10 and <30) were provisionally accepted into the trial.

The time of patient recruitment was 2 months prior to the start of the intervention. If, after two months, the 24 patients were not recruited, the intervention began with the available patients. Therefore, we prepared randomized series from 16 to 24, depending on the number of patients recruited.

At each PCC two nurses were responsible for carrying out patient assessment through an individual interview. These nurses were the same as those that would conduct the intervention group.

#### First evaluation and randomization

Initial assessment included sociodemographic characteristics (age, gender, nationality, marital status, educational level, number of children, employment status, economic status, core coexistence), medication (antidepressant, anxiolytics, hypnotics), administration of the Beck Depression Inventory scale (BDI) and the EuroQol- 5D (EQ-5D) questionnaire. All study variables were entered in a study database.

All patients evaluated who met the inclusion criteria were assigned a number consecutively. The participants were randomly allocated to one of two conditions by means of a computer-generated random allocation list. The computer program carried out series randomized from # 1 to 16, 18, 20, 22 and 24#, depending on the number of participants in each PCC. For the intervention group and control group the same number of patient*s* was used (minimum 16 people and maximum 24 per PCC).

An independent person was responsible for managing the randomization lists. Subsequently, this individual sent the randomization lists in a sealed envelope to the two nurses at each PCC a few days before the intervention began.

#### Follow up evaluations

All outcome variables were assessed four times: prior to start of the study (pretest), after 3 months (post-test), and at 6 and 9 months after inclusion (first and second follow-up, respectively) in individual data collection sessions.

### Measures

Diagnoses for participants were based on the International Classification of Diseases, 10^th^ revision (ICD-10) [40]. The diagnosis was made by the general practitioner. Prior to the use of questionnaires, permission was requested from the authors.

#### Beck depression inventory

The Beck Depression Inventory [41,42] is a brief scale of 21 items which assesses the severity of depression symptoms during the previous week. We selected the BDI due to its good internal consistency, validity, sensitivity to change, and the fact that it includes an assessment of cognitive and psychosocial symptoms.

The score range is 0–63 points. The usually accepted cut-off points for adjusting the intensity/severity are as follows: No Depression: 0–9 points, mild depression: 10–18 points, moderate depression: 19–29 points and severe depression: ≥ 30 points [43].

#### EuroQol quality of life questionnaire

The EQ-5D is a self-report scale allowing a multidimensional description of health and construction of a digital health profile. It is a standardized measure of health status, applicable to a wide range of health conditions and treatments which provides a simple descriptive profile and a single index value for health status [44]. This scale was validated in Spain by Xavier Badia in 1999 [45].

#### Remission

Clinical remission is based upon the BDI, which is a self-report screening instrument. Remission is defined as a mean BDI score of ≤11 [46]. On the BDI self-rating scale, a cut-off of BDI ≤11 emerged for remission with a sensitivity of 90% and specificity of 64%.

### Group treatments

#### Description of the training

Nurses who lead psychoeducational groups have received previous training in relation to depression (characteristic symptoms, diet, sleep, self-esteem, self-image, physical exercise and pharmacological treatment) and in the conducted-observation groups by therapists with extensive experience. They have also been trained in techniques such as problem solving, relaxation-breathing techniques, behavioral activation and cognitive restructuring therapy. The training period was 40 hours.

The GPs received previous training in relation to the detection and diagnosis of depression in patients, and the basic principles of group psychoeducational intervention.

#### Description of the psychoeducational group intervention

The intervention consisted of 12 weekly, 90 minute sessions led by two nurses. A total of 24 nurses collaborated in the study, two nurses per PCC. During the study period, twelve groups were formed. Each group consisted of 8–12 participants.

The research group developed a protocol with a program of 12 group sessions in order to homogenize the study interventions [47].

The description of the objectives of the 12 sessions is shown in Table 1. The program provided: 1. Health education about the illness: symptoms, diet, physical exercise, sleep, pharmacological treatment and adherence to treatment. 2. Breathing techniques. 3. Problem solving, Behavioral activation and Cognitive-behavioral perspective on depression. 4. Self-esteem and self-image. 5. Pleasant activities, social skills and assertiveness.

**Table 1:** Psychoeducational program

**Sessions**	**Objectives**
1	First contact with the group
	Breathing techniques
2	Behavioral Activation I
	Health education and identificated of depressive symptoms
3	Behavioral Activation II
4	How to take care to advance I
	- Diet
	- Sleep
	- Educational about pharmacological treatment
5	Problem solving I
6	Problem solving II
7	Self-esteem and self-image
8	Assertiveness
9	How to take care to advance II
	- Pleasant activities, social skills
	- Physical exercise
10	Cognitive-behavioral perspective I
11	Cognitive-behavioral perspective II
12	Group farewell
	Final evaluation

To enhance the active role of the patient, each session was accompanied with homework for the patient. The participants were free to continue under pharmacological treatment.

The group interventions were conducted on the PCC premises. All PCC settings had the space and equipment necessary to carry out the intervention.

#### Description of the control group

Members of the control group received usual treatment (visits with the GP and nurses). There was no pattern of visits established; the patients could go to the PCC when they needed to. The GPs and nurses use their own criteria to attend depressed patients. During the visits, the patient was asked about their general health status, adherence to antidepressant treatment (if they had prescribed medication) and the GP and nurses answered any queries about healthy lifestyle (such as sleep, diet and exercise). Each visit lasted 10 to 20 minutes. The participants were free to continue under pharmacological treatment.

### Analysis

#### Sample size calculation

The sample size was determined by practical restrictions and estimation of statistical power. Accepting an alpha risk of 5% and a beta risk of 20% in a bilateral contrast, at least 92 subjects were needed in the intervention group and 92 in the control to detect a difference equal to or greater than 4.5 units on the BDI scale. It is assumed that common standard deviation is 10. A loss rate of up 15% has been estimated.

#### Statistical analysis

The analysis was carried out on an intent-to-treat basis. The analyses were based on the data of the 231 participants who completed some of the evaluations. The intent-to-treat analysis was carried out as follows; missing values were replaced by the scale scores of the previous assessment (the last observation carried forward (LOCF)) to assure no increase. To examine baseline differences in the sociodemographic and clinical characteristics between groups, the t-Student test for continuous variables and Chi-square test for categorical variables were applied.

The effect of the intervention on the outcome variables was measured by the difference in scores between groups and the effect size. Standardized effect size (SES) [48] is calculated as the mean difference between the intervention and the control groups, divided by the standard deviation (SD) of the control group. The SES is a standardized measure of the change that allows comparison between groups, between measures in the same study and between different studies [49].

The standardized mean response (SMR) was used to measure the effect size within group comparisons. The SMR was calculated as the mean change divided by the SD of the change. Cohen’s d allows classification of effect size into small (0.2 to 0.5), medium (0.5 to 0.8) and large (0.8 or over); these criteria can also be applied to SMR [49,50]. The statistics package IBM SPSS Statistics v.18 was used [51].

To evaluate the evolution of the BDI between groups, we produced a mixed- effects model, using the monitoring time (4 times: pretest, 3-month, 6-month and 9-month) as a random effect, and as a fixed effect: the type of intervention (control versus intervention), age, gender and the type of PCC. We evaluated the goodness of fit through the Kolmogorov-Smirnov test of the model residuals.

## Results

### Participant flow

**Figure 1:**
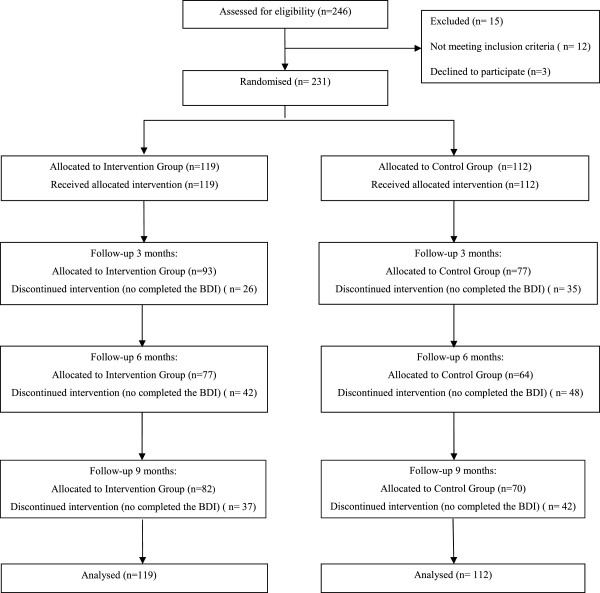
Flow chart of participants.

The flow of participants is shown in Figure 1. Of the 246 assessed in the study, 15 people declined to participate.

### Patients characteristics

231 patients were included in the study. Patients were randomized either to psychoeducational intervention (n=119) or control group (n=112). These two groups were similar at baseline in terms of demographic and clinical characteristics, except with respect to marital status (p=0.030). Table 2 shows the baseline characteristics of the total study population and the intervention group. The typical patient was a woman, Spanish national, approximately 54 years old, married/cohabitant, with primary education and self-employed. She had one/two children and referred to a stressful event in the previous months.

**Table 2:** Baseline characteristics of the total study population and intervention group. Values are numbers (percentages)

**Variable Category**	**Variable Category**	**General n (%)**	**Intervention n (%)**	**Control n (%)**
Gender	Women	206(89.2)	108 (90.8)	98 (87.5)
Age	Mean (SD)	53.38(12.63)	52.29(11.77)	54.54 (13.44)
Nationality	Spanish	215 (93.1)	111 (93.3)	104 (92.9)
	Single	33 (14.3)	20(16.8)	13 (11.7)
Marital status *	Married/cohabitant	119 (51.7)	65 (54.6)	54 (48.2)
	Divorced/separed	41 (17.8)	23 (19.3)	18 (16.2)
	Widow/widowed	37 (16.1)	11 (9.2)	26 (23.4)
	Not completed primary education	27 (11.8)	14 (11.8)	13 (11.9)
Educational level	Completed primary education	88 (38.6)	42 (35.3)	46 (42.2)
	Secondary education	69 (30.3)	39 (32.8)	30 (27.5)
	University	44 (19.3)	25 (20.8)	20 (18.3)
Nº Childrens	0 Children	63 (27.3)	31 (26.1)	32 (28.6)
	1-2 Children	113 (48.9)	61 (51.3)	52 (46.4)
	>=3Childrens	55 (23.8)	27 (22.7)	28 (25)
	Self- Employed	97 (42.4)	56 (47.1)	41 (37.3)
	Disability or permanent disability	20 (8.7)	9 (7.6)	11 (10)
Employment status	Unemployed	32 (14)	18 (15.1)	14 (12.7)
	Works at home	36 (15.7)	19 (16)	17 (15.5)
	Retired	44 (19.2)	17 (14.3)	27 (24.5)
	Alone	41 (17.9)	17 (14.2)	24 (21.8)
	With childrens	32 (14)	17 (14.3)	15 (13.6)
	With his/her partner	52 (22.7)	28 (23.5)	24 (21.8)
	With his/her partner and children	66 (28.8)	36 (30.3)	30 (27.3)
Core coexistence	With parents	13 (5.7)	8 (6.7)	5 (4.5)
	With others family	11 (4.8)	8 (6.7)	3 (2.7)
	With other people	8 (3.5)	3 (2.5)	5 (4.5)
	Others	6 (2.6)	2 (1.7)	4 (3.6)
	Permanent contract	74 (34.7)	43 (37.7)	31 (31.3)
Employment Economic status	Temporary contract	8 (3.8)	3 (2.6)	5 (5.1)
	Self-employment	14 (6.6)	7 (6.1)	7 (7.1)
	Work without contract	12 (5.6)	9 (7.9)	3 (3)
	Not work, but have a salary	77 (36.2)	39 (34.2)	38 (38.4)
	Not work, not salary	28 (13.1)	13 (11.4)	15 (15.2)
Stressful event	Yes	138 (63)	73 (65.2)	65 (60.7)
Medication: Antidepressant	Yes	129 (55.8)	71 (55)	58 (45)
	No	102 (44.2)	48 (47.1)	54 (52.9)
	SSRI	105 (45.7)	60 (50.4)	45 (40.5)
	Tricyclic	6 (2.6)	4 (1.7)	2 (0.9)
	Dual	20 (8.7)	9 (7.6)	11 (9.9)
Medication: Anxiolytics	Yes	125 (54.3)	67 (56.8)	58 (51.8)
Hypnotics	Yes	11 (4.8)	7 (5.9)	4 (3.6)
Alternative treatment	Yes	51 (22.1)	28 (23.5)	23 (20.5)
Medication: blood pressure	Yes	70 (30.3)	32 (26.9)	38 (33.9)

**Abbreviations: SD:** standard deviation; **SSRI:** serotonin-norepinephrine reuptake inhibitors.

* P value significant (p=0.030).

Those allocated to the psychoeducational group received a mean of 8.68 (SD 3.64; range 0–12) sessions. Adherence to psychoeducational intervention was reasonably good, with only 2 of 119 (1.68%) patients not attending any sessions and 88 (73%) receiving at least eight or more sessions.

The sessions received by the intervention group were: 12 sessions (n=38); 11 sessions (n= 15); 10 sessions (n= 23); 9 sessions (n= 6); 8 sessions (n= 6); 7 sessions (n= 3); 6 sessions (n= 3); 5 sessions (n= 2); 4 sessions (n= 1); 3 sessions (n=1); 2 sessions (n= 5); 1 session (n= 14) and 0 sessions (n= 2).

### Attrition and dropout

Sixty-one patients were not evaluated at post-treatment (did not respond to the BDI questionnaire), 26 from the IG and 35 from the CG. At 6 month follow up, 42 from the IG and 48 from the CG, and at 9 month follow up, 37 from the IG and 42 from the CG.

Of these, 61 patients were not evaluated at post-treatment, 54 of them were drop-outs (drop-outs = patients who were not evaluated at post-treatment and follow-up assessments at 6 and 9 months). Therefore, the overall drop-out rate was 23%. The drop-out rate was 19.3% (n=23) in the intervention group and 24.1% (n=27) in the control group. Drop-outs from the experimental condition did not differ statistically from those in the control group at follow-up assessment. The reasons for drop-out were: not contactable by telephone and did not attend the interview with the nurse (n=42), not interested in the study (n=1), change of address (n=3), referred to a secondary mental health service (n=2) and other unspecified reasons (n=6).

We analysed the 231 patients included in the study, as the analysis was carried out on an intent-to-treat basis.

### Intervention effectiveness: remission

The proportion of patients achieving remission status (BDI≤11 score) was examined using the Riedel remission criteria for major depression [46].

Post-test results showed that more participants in the intervention group (34.5%) had scored in the non-symptomatic BDI range (BDI≤11 score) than participants in the control group (18.8%); the 15.7% difference was statistically significant (p=0.003, 95% CI 4.5 to 26.9). After 6 and 9 month follow-up the results were similar; the proportion was 40.3% in the intervention group and 26.8% in the control group and the 13.5% difference was statistically significant (p=0.014, 95% CI 1.5 to 25.6). Table 3 shows the proportion of patients remitting through treatment in the overall, mild and moderate sample. The *number needed to treat (NNT)* is about 6.4 at short-term, and 7.4 for the long-term (after 9 months); i.e. reducing BDI below 11.

**Table 3:** Remission of depression in the overall, mild and moderate sample

**Sample**	**Months**	**Control n (%)**	**Intervention n (%)**	% difference at each follow-up *	**(IC95%)**	**P-value**
		**(n= 112)**	**(n= 119)**			
**Overall**	**3**	21 (18.75)	41 (34.45)	15.70	(4.5 to 26.9)	0.003
	**6**	30 (26.79)	48 (40.34)	13.55	(1.5 to 25.6)	0.014
	**9**	30 (26.79)	48 (40.34)	13.55	(1.5 to 25.6)	0.014
		**(n= 37)**	**(n= 48)**			
	**3**	15 (31.30)	21 (56.80)	25.50	(5.01 to 46)	0.009
**Mild**	**6**	20 (41.70)	22 (59.50)	17.80	(−3.3 to 39)	0.051
	**9**	18 (37.50)	24 (64.90)	27.40	(6.7 to 48)	0.006
		**(n= 82)**	**(n= 64)**			
	**3**	6 (9.40)	20 (24.40)	15.00	(2.7 to 27.2)	0.007
**Moderate**	**6**	10 (15.60)	26 (31.70)	16.10	(2.2 to 29.9)	0.011
	**9**	12 (18.80)	24 (29.30)	10.50	(− 3.4 to 24.5)	0.068

**Abbreviations: IC** interval coefficient.

* Difference was calculated between intervention and control group.

As we were interested in analyzing what kind of population can benefit most from receiving the intervention, participants were categorized into mild (BDI ≤18) and moderate initial depressive symptomatology (BDI ≥19), based on the pretest BDI sample median. Of the total sample of 231 people, 86 had mild symptoms and 146 moderate symptoms at baseline.

In patients with mild depression (pretest BDI), results showed that the remission was statistically significant at post-test and at 6 and 9 month follow-up.

Post-test results showed that the proportion in the non-symptomatic range of the BDI was 56.8% in the intervention group and 31.3% in the control group, the 25.5% difference was statistically significant (p=0.009, 95% CI 5.01 to 46). At 6 month follow-up, the proportion was 59.5% in the intervention group and 41.7% in the control group, but the 17.8% difference was not statistically significant (p=0.051, 95% CI −3.3 to 39). After 9 months the proportion was 64.9% in the intervention group and 37.5% in the control group; the 27.4% difference was statistically significant (p= 0.006, 95% CI 6.7 to 48) (Table 3).

In patients with moderate depression (pretest BDI), results showed that the remission was statistically significant at post-test and 6 month follow-up. At post-test, the proportion was 24.4% in the intervention group and 9.4% in the control group; the 15% difference was statistically significant (p=0.007, 95% CI 2.7 to 27.2) and at 6-month follow-up it was 31.7% in the intervention group and 15.6% in the control group (16.1% difference; p= 0.011, 95% IC 2.2 to 29.9) but after 9 months, results were not statistically significant (10.5% difference; p=0.068, 95% CI −3.4 to 24.5) (Table 3).

### Depressive symptomatology

Depressive symptoms were assessed through the Beck Depression Inventory (BDI). The difference between treatments at 3 months (psychoeducation intervention minus control) was estimated to be – 2.12 (95% CI −4.03 to −0.214) which was significant (P=0.029). The negative sign indicates that participants in the psychoeducation intervention had fewer depressive symptoms than those in the control group. The results at 6 and 9 months were not significant. Table 4 shows the changes in the BDI within and between the intervention and usual care group with missing data replaced using last value carried forward.

**Table 4:** Overall, mild and moderate sample. Changes in the BDI within and between the intervention and usual care group with missing data replaced using last value carried forward

			**Usual care group (n=112)**			**Intervention group (n=119)**		Difference (95% CI) between groups (intervention group -usual care group)**		
**Sample**	**Months**	**mean (SD)**	Difference* (95% CI)	**SRM**^**#**^	**mean (SD)**	Difference*(95% CI)	**SRM**^**#**^	**Difference**	**P-value**	**SES**^**$**^
	**Pre-intervention**	19.62 (5.79)			20.90 (5.68)					
	**3 (Post-intervention)**	17.54 (7.18)	2.07 (1.0 to 3.1)	0.36	15.42 (7.53)	5.47(4.19 to 6.76)	0.77	−2.12 (−4.03 to −0.214)	0.029	0.29
**Overal**	**6**	16.51 (7.60)	3.1 (1.7 to 4.4)	0.43	15.37 (8.74)	5.52 (3.9 to 7.08)	0.64	−1.13 (−3.27 to 0.992)	0.293	0.15
	**9**	16.35 (7.84)	3.26 (1.9 to 4.6)	0.44	15.09 (8.62)	5.8 (4.3 to 7.26)	0.72	−1.25 (−3.39 to 0.886)	0.249	0.16
			**Usual care group (n=48)**			**Intervention group(n=37)**				
	**Pre-intervention**	14.08 (2.72)			13.81 (2.50)					
**Mild**	**3 (Post-intervention)**	13.23 (5.57)	0.85 (−0.56 to 2.2)	0.17	10.38 (4.94)	3.43(1.81 to 5.04)	0.71	−2.85 (−5.16 to −0.542)	0.016	0.51
	**6**	13.15 (6.02)	0.93 (−0.78 to 2.65)	0.15	10.65 (5.46)	3.16 (1.15 to 5.16)	0.52	−2.50 (−5.015 to 0.200)	0.052	0.42
	**9**	12.27 (5.78)	1.81 (0.06 to 3.56)	0.30	9.70 (5.93)	4.11 (2.17 to 6.04)	0.70	−2.57 (−5.114 to −0.220)	0.048	0.44
			**Usual care group (n=64)**			**Intervention group(n=82)**				
	**Pre-intervention**	23.77 (3.6)			24.10 (3.31)					
**Moderate**	**3 (Post-intervention)**	20.8 (6.6)	2.99 (1.45 to 4.51)	0.49	17.7 (7.4)	6.40(4.71 to 8.09)	0.83	−3.08 (−5.41 to −0.762)	0.010	0.47
	**6**	19.00 (7.7)	4.74 (2.86 to 6.60)	0.63	17.5 (9.1)	6.60 (4.54 to 8.65)	0.70	−1.53 (−4.35 to 1.28)	0.285	0.20
	**9**	19.4 (7.8)	4.36 (2.35 to 6.36)	0.54	17.50 (8.6)	6.58 (4.63 to 8.50)	0.74	−1.89 (−4.60 to 0.840)	0.174	0.24

**Abbreviations: SD** standard deviation; **CI** confidence interval.

* Differences were calculated between baseline measurement and follow-up measurement.

Positive differences indicate improvement; a negative one denotes some worsening on clinical measures.

**# SRM: Standardized response mean**. Calculated as the mean change in score divided by the standard deviation of the change in score.

**$ SES: Standardized effect size** was computed as the mean difference between intervention and control group divided by the standard deviation of the control measurement.

A positive SRM or SES denotes improvement; a negative one denotes some worsening on clinical measures.

** Difference was calculated between intervention group and control group.

Negative differences indicate improvement in the intervention group; Positive differences denote worsening in the intervention group.

**Interpretation effect sizes**: Values 0.2-0.5 represent small changes, 0.5-0.8 moderate changes and >0.8 large changes.

**Figure 2:**
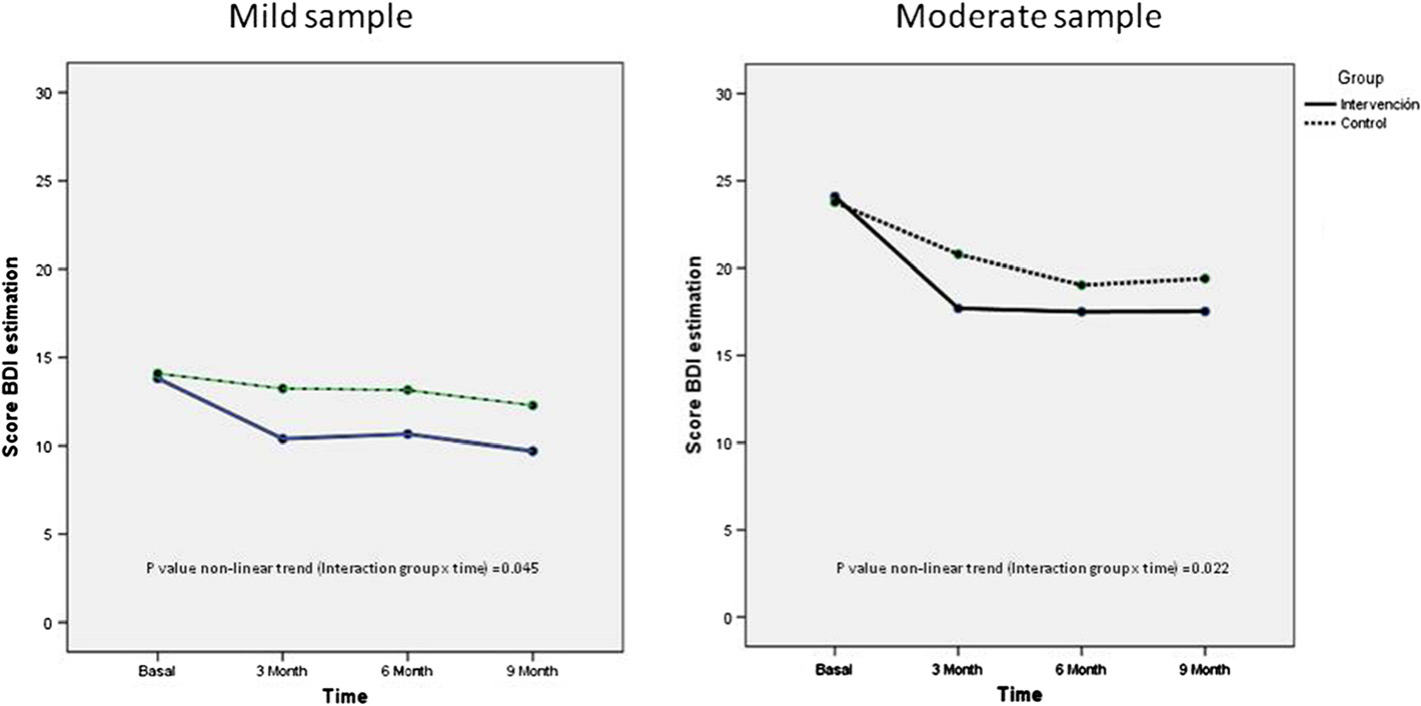
Evolution of the BDI over time in the overall sample.

A 2 (condition: intervention, control) x 4 (time: pretest, 3-month follow-up (post-test), 6-month follow-up, and 9-month follow-up) mixed-effects linear regression model showed a significant condition x time interaction. The results show that the evolution of the BDI over time between groups was significant, (p value non- linear trend =0.007). The effect size of this contrast is smaller in the short (post-test) and long term (9 month follow up): d’=.29 and d’=.16, respectively. Figure 2 shows the evolution of the BDI over time in the overall sample.

If, in this sample, we analysed the intervention and control group separately, the effect size within the intervention group (SRM) was moderate over time (d’= .77 post- intervention and d’=.72 at 9 month follow up) and the effect size within the control group was small over time (d’= .36 post- intervention and d’=.44 at 9 month follow up).

In patients with mild depression (pretest BDI), the difference between treatments at 3 months (psychoeducation intervention minus control) was estimated to be −2.85 (95% CI −5.16 to −0.542) which was significant (p=0.016). The results at 6 and 9 months were significant (p=0.052 and p=0.048, respectively) (Table 4). The results show that the BDI was significantly affected by the time, p<.001, indicating that self-reported depressive symptomatology declined significantly during the course of treatment although regardless of which treatment was received, it was significantly affected by the type of intervention, (p value no-linear trend =0.045). The effect size of this contrast is moderate at short-term (d’= .51) and at long-term (d’= .44).

If, in this sample, we analysed the intervention and control group separately, the effect size within intervention group was moderate over time (d’= .71 post-intervention and d’=.70 at 9 month follow up) and the effect size within control group was small over time (d’= .17 post- intervention and d’=.30 at 9 month follow up).

**Figure 3:**
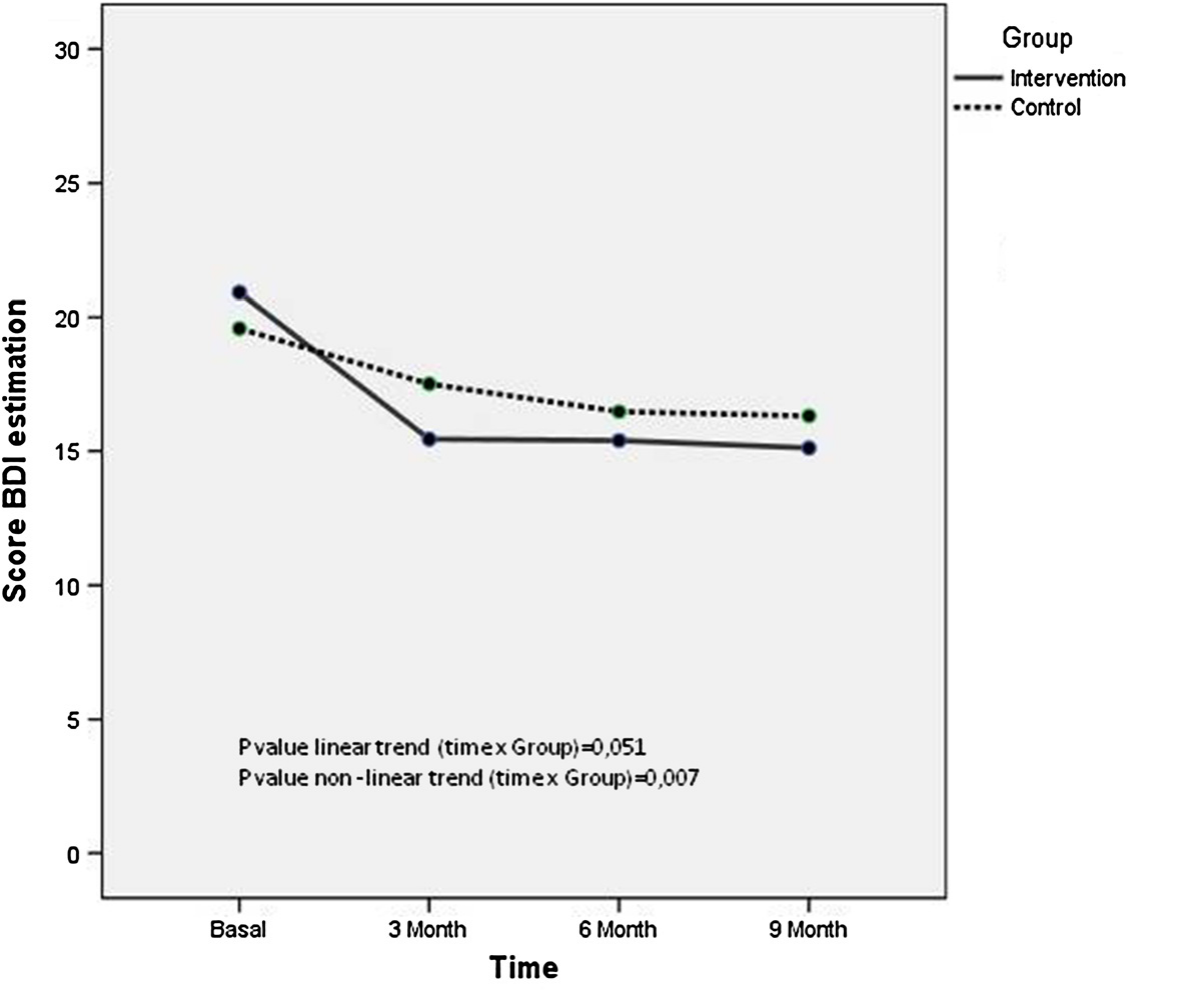
Evolution of the BDI over time in the mild and moderate sample.

In patients with moderate depression (pretest BDI), the difference between treatments at 3 months (psychoeducation intervention minus control) was estimated to be −3.08 (95% CI −5.41 to −0.76) which was significant (p=0.001). The results at 6 and 9 months were not significant (p=0.58 and p=0.17, respectively) (Table 4). The results show that the BDI was significantly affected over time, p<.001 and according to type of intervention, (p value non-linear trend =0.022). The effect size of this contrast is smaller in the short-term (d’=.47) and the long-term (d’= .24). Figure 3 shows the evolution of the BDI over time in the mild and moderate sample.

If, in this sample, we analysed the intervention and control group separately, the effect size within intervention group was large (d’= .83) post-intervention and moderate at 9 month follow up (d’=.74), and the effect size within control group was small (d’= .49) post-intervention and moderate (d’=.54) at 9 month follow up.

### Quality of life

Quality of life was assessed using the EQ-5D questionnaire. Table 5 shows the changes in the EQ-5D within and between the intervention and usual care group with missing data replaced using last value carried forward. The difference between treatments at 3 months (psychoeducation intervention minus control) was estimated to be 4.19 (95% CI −0.31 to 8.66) which was a trend towards significance (p=0.067). The positive sign indicates that participants in the psychoeducational intervention improved their quality of life more than the control group. The results at 6 and 9 months were not significant, and the difference between treatments descends at 9 months to 1.54 (95% CI −3.43 to 6.51). If, in this sample, we analyzed the intervention and control group separately, the post-intervention results show that the intervention group had an improvement in the EQ-5D of 8.97 points (95% CI 12.20 to 5.72; p<0.001) and was statistically significant. In the control group, the improvement was 2.29 points (95% CI 4.6 to −0.01; p=0.052), which was a trend towards significance (Table 5).

**Table 5:** Overall, mild and moderate sample. Changes in the EQ-5D within and between the intervention and usual care group with missing data replaced using last value carried forward

			**Usual care group (n=112)**			**Intervention group (n=119)**		Difference (95% CI) between groups (intervention group -usual care group)**		
**Sample**	**Months**	**mean (SD)**	Difference*(95% CI)	**SRM**^**#**^	**mean (SD)**	Difference*(95% CI)	**SRM#**	**Difference**	**P-value**	**SES**^**$**^
	**Pre-intervention**	53.25 (17.63)			50.76 (18.73)					
	**3 (Post-intervention)**	55.54 (16.36)	2.29 (4.6 to −0.01)	0.19	59.7 (18.1)	8.97(12.20 to 5.72)	0.50	4.19 (−0.31 to 8.66)	0.067	0.26
**Overall**	**6**	57.05 (16.97)	3.80 (6.98 to 0.61)	0.22	57.9 (20.7)	7.09 (10.78 to 3.39)	0.34	0.81 (−4.12 to 5.73)	0.748	0.05
	**9**	57.69 (17.35)	4.44 (8.0 to 0.87)	0.23	59.2 (20.8)	8.46 (11.99 to 4.93)	0.43	1.54 (−3.43 to 6.51)	0.543	0.09
			**Usual care group (n=48)**			**Intervention group (n=37)**				
	**Pre-intervention**	57.92 (18.10)			57.81 (17.58)					
**Mild**	**3 (Post-intervention)**	60.71 (16.00)	2.79 (6.17 to −0.59)	0.24	65.7 (16.7)	7.89(13.84 to 1.94)	0.44	4.99 (−2.11 to 12.09)	0.166	0.31
	**6**	60.90 (16.49)	2.98 (7.71 to −1.75)	0.18	64 (18.1)	6.14 (12.73 to −0.47)	0.31	3.05 (−4.43 to 10.53)	0.420	0.18
	**9**	62.52 (15.02)	4.60(9.75 to −0.54)	0.25	67.8 (20.5)	9.97 (16.82 to 3.11)	0.48	5.26 (−2.39 to 12.92)	0.175	0.35
			**Usual care group (n=64)**			**Intervention group (n=82)**				
	**Pre-intervention**	49.75 (16.56)			47.59 (18.46)					
	**3 (Post-intervention)**	51.67 (15.67)	1.92 (5.15 to −1.31)	0.15	57.04 (18.08)	9.45(13.39 to 5.50)	0.52	5.36 (−0.263 to 10.99)	0.062	0.34
**Moderate**	**6**	54.17 (16.88)	4.42 (8.82 to 0.02)	0.25	55.11 (21.35)	7.52 (12.07 to 2.97)	0.36	0.93 (−5.49 to 7.37)	0.774	0.06
	**9**	54.06 (18.20)	4.31 (89.33 to −0.70)	0.21	55.37 (19.83)	7.78 (11.95 to 3.60)	0.40	1.30 (−5.00 to 7.61)	0.684	0.07

**Abbreviations: SD** standard deviation. **CI** confidence interval.

* Differences were calculated between follow-up measurement and baseline measurement.

Positive differences indicate improvement; a negative one denotes some worsening on clinical measures.

**# SRM: Standardized response mean.** Calculated as the mean change in score divided by the standard deviation of the change in score.

**$ SES: Standardized effect size** was computed as the mean difference between intervention and control group divided by the standard deviation of the control measurement.

A positive SRM denotes improvement; a negative one denotes some worsening on clinical measures.

** Difference was calculated between intervention group and control group.

Positive differences indicate improvement in the intervention group. Negative differences denote worsening in the intervention group.

**Interpretation effect sizes:** Values 0.2-0.5 represent small changes, 0.5-0.8 moderate changes and >0.8 large changes.

Separate sample analyses in patients with mild depression showed the difference between treatments at 3 months (psychoeducation intervention minus control) was estimated to be 4.99 (95% CI −2.11 to 12.09) which was not significant (p=0.166). The results at 6 and 9 months were not significant, but the difference between treatments at 9 months was estimated to be 5.26 (95% CI −2.39 to 12.92), thus the improvement in quality of life was maintained over time. If, in this sample, we analyzed the intervention and control group separately, the post-intervention results show that the intervention group had an improvement in the EQ-5D of 7.89 points (95% CI 13.84 to 1.94; p=0.011), this was statistically significant and the improvement was maintained over time. The control group had an improvement of 2.79 points (95% CI 6.17 to 0.59; p=0.103), which was not significant.

In patients with moderate depression, the difference between treatments at 3 months (psychoeducation intervention minus control) was estimated to be 5.36 (95% CI −0.26 to 10.99) which was not significant (p=0.062). The results at 6 and 9 months were not significant. If, in this sample, we analyzed the intervention and control group separately, the post-intervention results show that the intervention group had an improvement in the EQ-5D of 9.45 points (95% CI 13.39 to 5.50; p<0.001). This was statistically significant, and the Control Group showed no significant improvements; -1.92 points (95% CI 5.15 to 1.31; p=0.239).

## Discussion

We found a relationship between the psychoeducational group intervention and remission of depressive symptoms. More patients from the IG had remission of their depressive symptoms at short and long term compare with the control group. The psychoeducational group intervention proved to be effective in the short term, showing a reduction in the BDI score of 5 points and this symptomatic improvement in the BDI continued to follow up at 9 months. In contrast, the control group needed 9 months to achieve a 3 point improvement in the BDI. We could say that it is an effective intervention in the short term, although the effect size is small.

When analyzing what kind of population can benefit most from receiving the group intervention; mild or moderate depression participants, we found that patients with mild symptoms obtained a higher rate of symptom remission in the short and long term and symptomatic improvement in the BDI remained over the long term, as distinct from the moderate depression group, where the improvement was only significant post-intervention (short term).

### Comparison with other studies

Psychoeducation has proved effective as psychotherapy for depressive-symptom management in the Primary Care setting [24,26-28,30,52,53]. However, there is a need to clarify both the magnitude of the effect (from 0.21 to 0.80) of this intervention and the associated factors that influence the measurement of efficacy. Some factors to take into account have been described: the type of psychoeducational intervention used [24,26,27,29,30,53], the clinical rating scale [24,26,27,30,53], intensity of clinical depressive symptoms at the beginning [26,28,54], and the duration of the therapeutic effect of the intervention [24,26-28,30,52].

One of the greatest difficulties in reviewing studies on the effectiveness of group psychoeducation in depression in primary care is that there is no consensus on the definition of psychoeducation. The studies which have used the term "psychoeducation" to define the type of psychological therapy have defined it as an applied educational-behavioral intervention [29], or interventions with behavioral components (behavior change, pleasant activities), cognition (cognitive restructuring, counseling), education (direct instruction, lectures) and competence (broad skill training) [26,55]. Thus, we find interventions of various orientations that share a high didactic and psychoeducational group structure. This would include CBT orientation interventions with a psychoeducational group format structure [53], specific psychoeducational interventions to improve adherence to drug treatment [29], and multicomponent interventions (stepped care) structured in a psychoeducational group format [30]; or the CWD course of cognitive-behavioral orientation [14,24,26,27,31]. Most studies [24,27,28,30,52,53] that have used a psychoeducational intervention also included homework for the patient.

In our study, we developed a psychoeducational group intervention protocol that included material from the 12 sessions [47], a CD with the material from each session and homework for the patient; so reaffirming the concept of psychoeducation in our intervention.

There are few randomized studies of group psychoeducation based on this approach of providing education about the disorder and healthy lifestyle behaviors; aspects that have been shown to help in the recovery of these patients [15,39,56].

Most studies that have evaluated the effectiveness of psychoeducation have used the Beck Depression Inventory (BDI) [24,26,27,52,53] as a clinical assessment scale, which, unlike the Hamilton Rating Scale for Depression (HRSD) [30], includes the psychological and psychosocial aspects, emphasizing the cognitive component of depression which is a very important issue in primary care.

The effectiveness of psychoeducation in the short term (post-intervention or 3 months) has already been demonstrated [26,30,52] and there are no discrepancies between studies. However, with respect to the duration of the therapeutic effect of psychoeducation at 6 and 12 month follow-up, results are controversial. The study by Allart-van Dam [28] evaluated the long-term preventive effects of an effective CWD course in the same sample of patients as in the earlier study [26] with a significant effect at 6 and 12 month follow-up (p=0.003 and p=0.03 respectively) but only in the participants with low initial symptomatology (BDI between 10 and 25). The study by Dalgard [27] evaluated the effect of a modified CWD course on unipolar depression at 6 and 12 month follow-up. Results showed that there was a significant improvement in symptoms (p=0.009) and the effect size at 6 month follow-up was small (d’=.47) but at 12 month follow-up, the BDI in the intervention group remained stable with a difference of 8 points. The study by Dowrick [26] evaluated the psychoeducation group for depressed adults and the results show us that the psychoeducation intervention reduced the severity and duration of depressive disorder after 6 months, but not at 12 months. In the study by Brown [52], patients significantly improved their BDI at 6 and 12 month follow-up, and in the study by Araya [30], significant improvements of 9 points between groups (p <0.0001) were maintained at 6 month follow-up.

Our results shows a significant improvement in symptoms post-intervention (p = 0.029; d’=.29) although the effect size is small but this improvement is not maintained at 9 month follow up (p = 0.24; d’=.16).

If we focus on the severity of initial clinical depressive symptoms, most studies included a sample of patients with an average BDI of 22. When we tried to identify the subgroup of patients which most benefits from this intervention, we divided the population according to their initial BDI: "mild" (BDI ≤ 18) or "moderate" (BDI ≥ 19). It was observed that patients with mild depressive symptoms have significantly improved symptoms in the short and long term (p = 0.001 and p = 0.048, respectively) with a moderate and small effect size (d’=.51 and d’=.44, respectively).

Our results coincide with those found in a review [54] where it is shown that psychological treatments for minor depression, including psychoeducation, are effective in the short-term (d’ = .42). However, the long-term improvement was not significant. Another study [26,28] concluded that patients with mild symptoms had low levels of depressive symptoms during the follow-up period, and at one year this population had depression scores that indicated an absence or very low level of symptoms.

However, when analyzing the sample of patients with moderate symptoms, our results show significant improvement in symptoms only in the short-term with a small effect size (d’ = 0.47).

In conclusion, we could say that the effectiveness of psychoeducation would be short-term (post-intervention) in the population with more severe symptomatology while, in patients with mild symptoms, it would be effective at both short and long term (follow-up at 6 and 9 months).

One of the most important aspects of our study is to evaluate the effectiveness of the intervention based on the remission of symptoms.

In relation to the remission of depressive symptoms (BDI ≤ 11) [46], our results show that 35% of the intervention group (IG) versus 19% in the control group (CG) had depressive symptoms post-intervention, and this 16% difference between groups was significant (p = 0.003). Follow-up at 6 and 9 months showed a significant difference of 14% (p = 0.01), with a 40% improvement in the IG versus 27% in the CG. These data are consistent with those found in one study [26] which showed that 52.5% of the intervention group did not present depressive symptoms (BDI <10) (Beck, 1988) post-intervention compared with the control group; 31.7%, with a significant difference of 20.8% (p = 0.04) between groups.

When analyzing the sample of patients with mild symptoms (initial BDI ≤ 18), we observed that 57% of the intervention group showed an absence of depressive symptoms versus 31% in the control group post-intervention, with a significant difference between groups of 25% (p = 0.009).

At 6 month follow-up, 59% of the intervention group improved versus 42% of the control group; a difference of 18%, and at 9 month follow-up, 65% improved in the intervention group versus 38% in the control group, with a 27% significant difference between groups (p = 0.006). These results support the effectiveness of this intervention in this subgroup of patients.

When we talk about the remission of symptoms in terms of number needed to treat (NNT), we observed that our NNT of 6.4 post-intervention and 7.4 at 9 month follow-up are supported by those obtained in the study by Dalgard [27] with a smaller sample of patients (n = 155) which was 6 at 6-month follow-up, and the Dowrick ODIN study [16] (n = 452) which was 7 at 6 months, supporting the effectiveness of the intervention. Another variable analyzed was quality of life and whether this could be associated with an improvement in depressive symptoms. At first, it was seen that the patients with mild symptoms have better quality of life compared with those with the most severe symptoms, although it has been observed at baseline that milder symptom patients already had better quality of life compared with those with the most severe.

Our results show that psychological intervention improves quality of life for both groups in the short-term, but only the patients with mild symptoms maintain this long-term improvement. No significant differences were found between the intervention group and the control group but this may be due to methodological issues related to the questionnaire used, EQ-5D, which has no cut-off points.

According to the results, this intervention is effective in both the short and long term with high rates of remission in patients with mild depressive symptoms.

We should mention the minor depression has a prevalence of 5–16% in primary care patients [57] and is an important risk factor for major depression, which develops in 10–25% of patients with subthreshold depression within 1–3 years [58]. It is also associated with psychological suffering, significant decrements in health, impairment in daily living activities and has a considerable impact on quality of life [57].

### Strengths and limitations of this study

Our trial has a number of strengths: firstly, it is the first study to assess the effectiveness of this psychoeducational group intervention which includes health education about the disorder, healthy behaviors, social skills and cognitive-behavioral techniques. Secondly, determining the target population; in this case patients with mild depression. Third, the study was conducted in Spain, specifically in Catalonia, and this is the first multicenter randomized study that assesses the effectiveness of a psychoeducational intervention in this country. Fourth, the sample population was representative of all Barcelona. The PCC participants were located in various areas of Barcelona, with different socio-demographic and economic resources. And finally, highlighting the role of the nurses who led the psycho-educational groups.

Despite the positive findings, potential biases need to be considered when evaluating the study. Some of the limitations of the study could be as follows: firstly, we performed a randomization of patients, but there is no double-blind, the patient knows who belongs to the intervention or control group, as do the nurses and doctors in the PCC. It was difficult for researchers to remain masked to group allocation. However, participants completed self-rating assessments of mood and quality of life. Therefore, that lack of blindness should not have affected our primary outcome to any great extent.

Secondly, the study employed only a two-outcome measure, BDI and EQ-5D, as we wanted the study to be as close as possible to the usual practice of the Primary Care Centers. It is a naturalistic study. Thirdly, the remission of depression was assessed by a screening questionnaire (BDI) rather than a diagnostic interview. Fourth, the overall drop-out was 23%, when we estimated a loss rate up 15%. This loss rate would affect to estimate the real evolution of the BDI a long-term. There wasn’t difference between groups in the loss rate. These losses are consistent with those found in other studies; between 25% and 37% [14,18]. Finally, further studies are required to confirm these results.

## Conclusions

Our results show that this psychoeducational intervention is more effective in patients with mild symptoms, since they have higher remission rate of symptoms at short and long term. Moreover, this improvement is associated with a better quality of life. The data do not demonstrate that the intervention is effective at long term in patients with moderate symptoms.

## Abbreviations

CI: confidence interval; d’: Cohen’s Effect size; IG: intervention group; CG: control group; ITT: intention to treat; NNT: numbers needed to be treated; PC: Primary care; PCC: primary care center; SES: Standardized effect size; SMR: standardized mean response.

## Competing interests

All authors declare that they have no competing interests.

## Authors’ contributions

RC designed the study, participated in the analysis and interpretation of data, wrote the manuscript and gave final approval of the version to be published. R Catalán was involved in drafting and revising the manuscript, and participated in the interpretation of data. JLV performed the statistical analysis and participated in the revision of the manuscript. JR performed the statistical analysis. SV participated in the design of the study. MC participated in the design of the study and in the revision of the manuscript. All authors contributed to the article and approved the final manuscript.

## Authors’ informations

RC is Psychologist. Research Department at the “Centre Higiene Mental (CHM) Les Corts”, Barcelona, Spain. Psychiatry and Legal Medicine Department, Universidad Autónoma de Barcelona, Spain. Barcelona Research Support Unit in Primary Care. IDIAP Jordi Gol, Catalan Institute of Health, Barcelona, Spain.

R Catalán is PhD Psychiatrist. Clinical Institute of Neurosciences (ICN), Hospital Clinic, Barcelona, Spain. Department of Psychiatry and Clinical Psychobiology, University of Barcelona, Spain. Institut d'Investigacions Biomèdiques August Pi i Sunyer (IDIBAPS), Barcelona, Spain. Centro de Investigación Biomédica en Red de Salud Mental (CIBERSAM), Spain.

JLV is a preventive public-health physician. Barcelona Research Support Unit in Primary Care. IDIAP Jordi Gol. Catalan Institute of Health Barcelona, Spain.

JR is a statistician. Barcelona Research Support Unit in Primary Care. IDIAP Jordi Gol. Catalan Institute of Health Barcelona, Spain.

SV is PhD Psychologist. Department of Psychiatry Hospital Universitari Vall d’Hebron, Barcelona, Spain. Centro de Investigación Biomédica en red de Salud Mental CIBERSAM, Spain.

MC is PhD Psychiatrist. Department of Psychiatry. Hospital Universitari Vall d’Hebron, Barcelona, Spain. Centro de Investigación Biomédica en red de Salud Mental CIBERSAM, Spain. Professor at the Universidad Autónoma de Barcelona, Spain.

## Pre-publication history

The pre-publication history for this paper can be accessed here:

http://www.biomedcentral.com/1471-244X/12/230/prepub
